# Glaucoma Is Not Associated With Alzheimer's Disease or Dementia: A Meta-Analysis of Cohort Studies

**DOI:** 10.3389/fmed.2021.688551

**Published:** 2021-08-24

**Authors:** Wenmei Zhao, Xia Lv, Guangjie Wu, Xia Zhou, Helan Tian, Xiang Qu, Hongpeng Sun, Yingying He, Yingyue Zhang, Chuan Wang, Jinyong Tian

**Affiliations:** ^1^Zunyi Medical University, Zunyi, China; ^2^Department of Emergency, Guizhou Provincial People's Hospital, Guiyang, China

**Keywords:** glaucoma, Alzheimer's disease, dementia, cohort studies, meta-analysis

## Abstract

**Background:** Previous studies evaluating the relationships of glaucoma with Alzheimer's disease (AD) and dementia showed inconsistent results. We performed a meta-analysis of cohort studies to evaluate the association between glaucoma with incidence of AD, all-cause dementia, and non-AD dementia.

**Methods:** Cohort studies which evaluated the association between glaucoma with incidence of AD, all-cause dementia, and non-AD dementia in adult population with multivariate analyses were identified by systematic search of PubMed, Embase, and Cochrane's Library databases. A random-effects model incorporating the potential intra-study heterogeneity was used for the meta-analysis.

**Results:** Eleven cohort studies including 4,645,925 participants were included. Results showed that compared to those without glaucoma at baseline, adult patients with glaucoma was not independently associated with increased incidence of AD [adjusted risk ratio (RR): 1.03, 95% confidence interval (CI): 0.93–1.05, *P* = 0.55; *I*^2^ = 83%], all-cause dementia (adjusted RR: 1.08, 95% CI: 0.97–1.19, *P* = 0.15; *I*^2^ = 79%), or non-AD dementia (adjusted RR: 1.05 95% CI: 0.91–1.21, *P* = 0.49; *I*^2^ = 82%). Sensitivity analyses by excluding one study at a time did not significantly affect the results of the meta-analyses. Moreover, subgroup analyses showed consistent results in meta-analysis of prospective or retrospective cohort studies, and in meta-analysis of patients with primary open-angle glaucoma or primary angle-closure glaucoma (*P*-values for subgroup difference all > 0.05).

**Conclusions:** Current evidence from cohort studies did not support that glaucoma is an independent risk factor of AD, all-cause dementia, or non-AD dementia in adult population.

## Background

Currently, Alzheimer's disease (AD) has become the most common cause of dementia for global population, which has significantly impaired the activities of daily living as well as the quality of life in these patients (1, 2). Despite of continuous efforts in the development of novel treatments, therapeutic strategies for patients with AD, as well as those with dementia of other causes, remain limited, and the prognosis of patients with AD are still poor (3–5). With the accelerated aging process of global population, early identification of populations that are of higher risk for AD and dementia is important for the prevention of the diseases (6–8).

Glaucoma is a group of neurodegenerative optic diseases which could affect populations with various age groups and lead to irreversible blindness (9). Moreover, glaucoma is not rare, with an estimated prevalence of 3.5% in people aged over 40 years (10, 11). Previous experimental studies showed that glaucoma and AD have common pathophysiological characteristics of neurodegeneration, such as reduced number of retinal ganglion cells, deposition of amyloid-β peptides, overactivated neuroinflammation, accumulation of tau proteins, and impaired insulin receptor signaling pathways (12–14). Besides, genetic studies showed that ε4/ε4 genotype of Apolipoprotein E (*ApoE*) may represent genetic overlaps between AD and glaucoma (15). Accordingly, epidemiological studies have suggested that glaucoma may be associated with higher odds of AD. In a previous meta-analysis of observational studies, Xu et al. included five cohort studies and three case-control studies and showed that patients with glaucoma were associated with higher risk of AD (16). However, the findings were mainly driven by the case-control studies included (17–19), which exposes the results to higher risk of patient-selection bias and recall-bias. In addition, studies with univariate or multivariate analyses were both included, which implied that the association may be potentially affected by the unadjusted confounding factors. Moreover, potential associations between glaucoma and the risks of all-cause and non-AD dementia have not been reported in the previous meta-analysis. Since some related cohort studies have been published after the previous meta-analysis (20–22), we aimed to perform an updated meta-analysis of cohort studies to evaluate the potential independent associations between glaucoma and AD, all-cause dementia, and non-AD dementia. Besides, potential influences of study design and subtypes of glaucoma on the outcomes were also explored in subgroup analyses.

## Methods

The meta-analysis was performed in accordance with the MOOSE (Meta-analysis of Observational Studies in Epidemiology) (23) and Cochrane's Handbook (24) guidelines.

### Literature Search

Studies were identified via systematic search of electronic databases of PubMed, Embase, and Cochrane's Library databases via the combination of the following terms: (1) “glaucoma”; and (2) “Alzheimer” OR “Alzheimer's” OR “dementia” OR “cognitive decline” OR “cognitive impairment” OR “cognitive dysfunction.” The search was limited to human studies published in English. The reference lists of related original and review articles were also analyzed using a manual approach. The final literature search was performed on October 13, 2020.

### Study Selection

The inclusion criteria for the studies were: (1) cohort studies published as full-length articles; (2) included general adult population; (3) evaluated the association between glaucoma with AD, all-cause dementia, and non-AD dementia; and (4) reported the risk ratios (RRs) for the above associations analyzed with multivariate adjustment at least for age and sex of the participants, or these data could be calculated. Diagnoses of glaucoma, AD, dementia, and non-AD dementia were in accordance with the criteria used in the original articles. Reviews, editorials, preclinical studies, and studies irrelevant to the aim of current meta-analysis were excluded.

### Data Extracting and Quality Evaluation

Literature search, data extraction, and quality assessment of the included studies were performed according to the predefined inclusion criteria by two independent authors. Discrepancies were resolved by consensus. The extracted data included: (1) name of first author, publication year, and country where the study was performed; (2) study design characteristics; (3) participant characteristics, including health status, sample size, age, and sex; (4) methods for the diagnosis of glaucoma and patients with glaucoma at baseline; (5) follow-up durations; (6) methods for validation of outcomes including incidence of AD, all-cause dementia, and non-AD dementia, and outcomes reported in each study; and (7) potential confounding factors adjusted in the multivariate analyses. The quality of each study was evaluated using the Newcastle-Ottawa Scale (25), which ranges from 1 to 9 stars and judges each study regarding three aspects: selection of the study groups; the comparability of the groups; and the ascertainment of the outcome of interest.

### Statistical Analyses

We used RRs and their corresponding 95% confidence intervals (CIs) as the general measure for the association between glaucoma and incidence of AD, all-cause dementia, and non-AD dementia. Data of RRs and their corresponding stand errors (SEs) were calculated from 95% CIs or *P*-values, and were logarithmically transformed to stabilize variance and normalized the distribution (24). The Cochrane's Q test and estimation of *I*^2^ statistic were used to evaluate the heterogeneity among the included cohort studies (26). A significant heterogeneity was considered if *I*^2^ > 50%. We used a random-effects model to synthesize the RR data because this model is considered as a more generalized method which incorporates the potential heterogeneity among the included studies (24). Sensitivity analyses, by omitting one individual study at a time, were performed to test the robustness of the results (27). Predefined subgroup analyses were performed to evaluate the influences of study characteristics on the outcome, including study design (prospective or retrospective) and subtypes of glaucoma, including primary open-angle glaucoma (POAG) or primary angle-closure glaucoma (PACG). The potential publication bias was assessed by visual inspection of the symmetry of the funnel plots, as well as the Egger's regression asymmetry test (28). We used the RevMan (Version 5.1; Cochrane Collaboration, Oxford, UK) and STATA software for the meta-analysis and statistics.

## Results

### Literature Search

**Figure 1:**
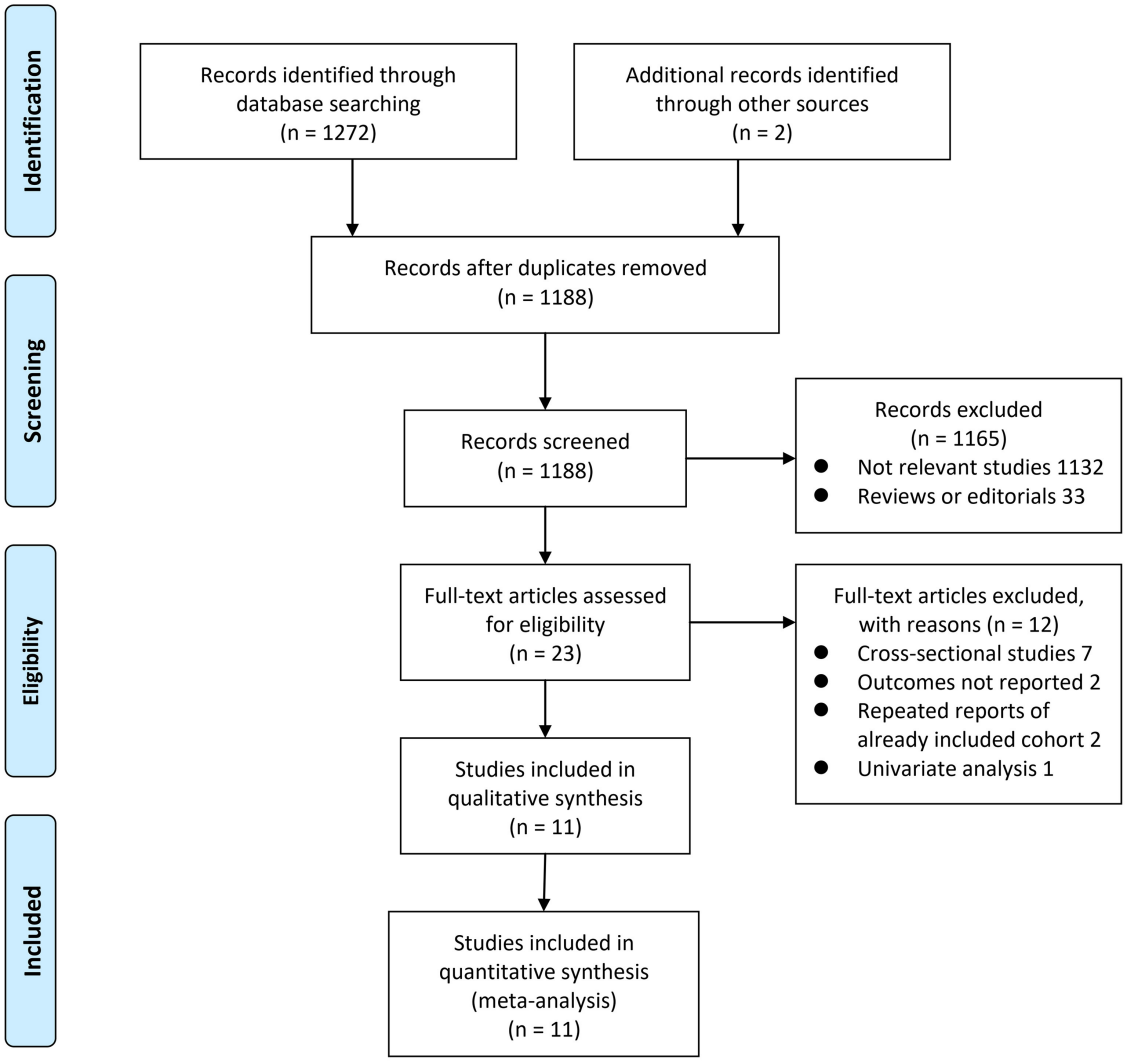
Flowchart of database search and study screening.

The process of database search was summarized in Figure 1. Briefly, 1,188 articles were found via initial literature search of the PubMed, Embase, and Cochrane's Library databases after excluding of the duplications. Among them, 1,165 were excluded through screening of the titles and abstracts mainly because they were not relevant to the purpose of the meta-analysis. Subsequently, 23 potential relevant records underwent full-text review. Of these, 12 were further excluded for the reasons listed in Figure 1. Finally, eleven observational studies were obtained for the meta-analysis (20–22, 29–36).

### Study Characteristics and Quality Evaluation

The characteristics of the included studies were summarized in Table 1. Overall, eleven cohort studies including 4,645,925 participants were included in the meta-analysis (20–22, 29–36). These studies were published between 2007 and 2020, and were performed in the United States (20, 30), United Kingdom (33), Denmark (29), Sweden (35), France (31), South Korea (36), and China (21, 22, 32, 34), respectively. Regarding study design, eight of them were retrospective cohort studies, while the remaining three were prospective cohort studies. All of the studies included adult populations. The sample size of the included studies varied between 812 and 2,623,130. The mean ages of the included participants varied from 59 to 80 years, and the proportions of male participants ranged from 35 to 53%. Glaucoma was diagnosed with eye examination in two studies (31, 35), with medical chart review in one study (22), and with international or national disease classification codes in eight studies (20, 21, 29, 30, 32–34, 36). The follow-up durations varied from 3 to 16 years. Outcomes of AD and dementia were diagnosed with the Diagnostic and Statistical Manual of Mental Disorders, Fourth Edition (DSM-IV) or the National Institute of Neurological and Communicative Disorders and Stroke-Alzheimer's Disease and Related Disorders Association (NINCDS-ADRDA) criteria in three studies (20, 22, 31), with medical chart evaluation in one study (35), and with disease classification codes in seven studies (20, 22, 31, 35). Age, sex, ethnicity, education, comorbidities, and other potential confounding factors were adjusted to a varying degree when the associations between glaucoma with incidence of AD, all-cause dementia, and non-AD dementia were reported. The NOS scores of the included studies ranged from seven to nine, indicating generally good study quality (Table 2).

**Table 1:** Characteristics of the included cohort studies.

**References**	**Country**	**Design**	**Patient characteristics**	**Number of patients**	**Mean age (years)**	**Male (%)**	**Patients with glaucoma**	**Glaucoma diagnosis**	**Follow-up duration (years)**	**Outcome validation**	**Outcomes reported**	**Variables adjusted**
Kessing et al. (29)	Denmark	RC	Adult population from National Hospital Register	410,544	68.2	37.1	17,696	ICD-8 or ICD-10 codes	4.6	ICD-8 or ICD-10 codes	AD, all-cause dementia, and non-AD dementia	Age at baseline, sex, time from discharge, and a diagnosis of substance use
Ou et al. (30)	the US	RC	Elderly people in a nationally representative sample	484,542	78.5	36	63,325	ICD-9 codes	14	ICD-9 codes	AD, and all-cause dementia	Age, sex, ethnicity, and CCI
Helmer et al. (31)	France	PC	Elderly people aged 72 years or older	812	79.7	35	41	IOP measurement or use of IOP lowering medications	3	DSM-IV criteria	All-cause dementia	Age, sex, education, hypertension, diabetes, history of cardiovascular ischemic disease, history of stroke, familial history of glaucoma, and APOE ε4
Lin et al. (32)	China	RC	Elderly people aged 60 years or older	19,895	71.3	52.9	3,979	ICD-9 codes	8	ICD-9 codes	AD	Age, sex, comorbidities (hypertension, diabetes, heart failure, stroke), insurance eligibility group, monthly income, diagnostic year, urbanization level and CCI;
Keenan et al. (33)	UK	RC	Elderly people aged 55 years or older	262,3130	NR	45.9	87,658	ICD-10 codes	NR	ICD-10 codes	AD, and non-AD dementia	Age, sex, calendar year of admission, region of residence and socioeconomic status
Su et al. (34)	China	RC	Adult population	32,545	59.2	45.5	2,609	ICD-9 codes	11	ICD-9 codes	All-cause dementia	Age, sex, hypertension, diabetes, CAD, hyperlipidemia, and head injury
Ekstrom and Kilander (35)	Sweden	RC	Elderly people aged 65~74 years	1,533	NR	NR	NR	Eye examination with IOP measurement	NR	Medical chart evaluation	AD	Age, sex, participating in the population survey, smoking, diabetes mellitus, hypertension, and ischaemic heart disease
Moon et al. (36)	South Korea	RC	Adult population from National Health Insurance Database	102,5340	NR	52.9	1,469	KCD codes	10	KCD codes	AD	Age, sex, residential area, income, CCI, hypertension, diabetes mellitus, hyperlipidemia and ischemic stroke
Lee et al. (20)	the US	PC	Elderly people aged 65 years or older	3,877	NR	42	404	ICD-9 codes	8	DSM-IV and NINCDS-ADRDA criteria	AD, all-cause dementia, and non-AD dementia	Age at enrollment, sex, education, ethnicity, APOE ε4 alleles, and smoking status
Kuo et al. (21)	China	RC	Adult people aged 20 years or older	42,048	NR	50	21,024	ICD-9 or ICD-10 codes	16	ICD-9 or ICD-10 codes	AD, all-cause dementia, and non-AD dementia	Age, sex, education level, marital status and related comorbidities
Xiao et al. (22)	China	PC	Elderly people aged 60 years or older from community	1,659	71.5	45.8	42	Medical records validation	5.2	DSM-IV and NINCDS-ADRDA criteria	AD, and all-cause dementia	Age, sex, education year, APOE ε4, baseline MMSE, smoking, alcohol consumption, hypertension, diabetes mellitus, BMI, depression, and heart disease

*RC, retrospective cohort; PC, prospective cohort; ICD, international classification of diseases; KCD, Korean classification of diseases; IOP, intraocular pressure; DSM-IV, the diagnostic and statistical manual of mental disorders, Fourth Edition; NINCDS-ADRDA, the national institute of neurological and communicative disorders and stroke-Alzheimer's disease and related disorders association; AD, Alzheimer's disease; CCI, the charlson comorbidity index; CAD, coronary artery disease; BMI, body mass index; MMSE, he mini-mental state examination*.

**Table 2:** Details of quality evaluation via the newcastle-ottawa scale.

**Study**	**Representativeness of the exposed cohort**	**Selection of the non-exposed cohort**	**Ascertainment of exposure**	**Outcome not present at baseline**	**Control for age and sex**	**Control for other confounding factors**	**Assessment of outcome**	**Enough long follow-up duration**	**Adequacy of follow-up of cohorts**	**Total**
Kessing et al. (29)	1	1	1	0	1	0	1	1	1	7
Ou et al. (30)	1	1	1	1	1	0	1	1	1	8
Helmer et al. (31)	0	1	1	1	1	1	1	1	1	8
Lin et al. (32)	1	1	1	0	1	1	1	1	1	8
Keenan et al. (33)	1	1	1	0	1	0	1	1	1	7
Su et al. (34)	1	1	1	1	1	1	1	1	1	9
Ekstrom and Kilander (35)	0	1	1	1	1	1	1	1	1	8
Moon et al. (36)	1	1	1	1	1	1	1	1	1	9
Lee et al. (20)	1	1	1	1	1	1	1	1	1	9
Kuo et al. (21)	1	1	1	1	1	1	1	1	1	9
Xiao et al. (22)	1	1	1	1	1	1	1	1	1	9

### Association Between Glaucoma and AD

**Figure 2:**
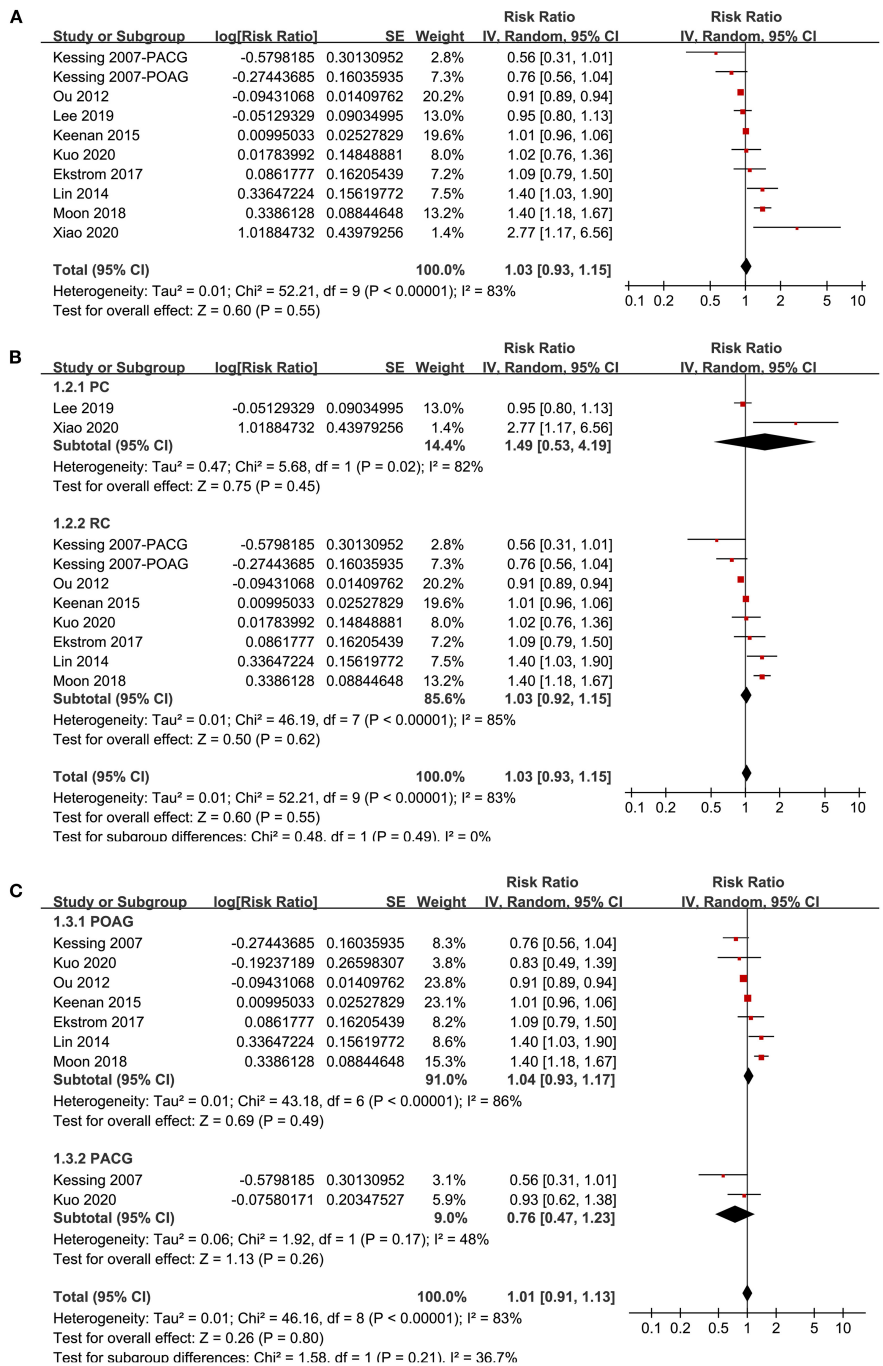
Forest plots for the meta-analysis of the association between glaucoma and the incidence of AD in adult population; **(A)** overall meta-analysis; **(B)** subgroup analysis according to the study design; and **(C)** subgroup analysis according to the type of glaucoma.

Nine studies (20–22, 29, 30, 32, 33, 35, 36) reported the association between glaucoma and incidence of AD in adult population. Since one study (29) reported the associations of POAG and PACG separately, these two datasets were included independently into the meta-analysis. Results of meta-analysis including ten datasets from nine studies showed that adult patients with glaucoma was not independently associated with an increased incidence of AD (adjusted RR: 1.03, 95% CI: 0.93–1.05, *P* = 0.55; Figure 2A) with significant heterogeneity (*P* for Cochrane's *Q*-test < 0.001, *I*^2^ = 83%). Sensitivity analyses by excluding one study at a time showed consistent results (*P* all > 0.05). Subgroup analyses showed that glaucoma was not independently associated with an increased incidence of AD in prospective (RR: 1.49, 95% CI: 0.53–4.19, *P* = 0.45; *I*^2^ = 82%) or in retrospective cohort studies (RR: 1.03, 95% CI: 0.92–1.15, *P* = 0.55; *I*^2^ = 85%; *P* for subgroup difference = 0.49; Figure 2B), or in studies of patients with POAG (RR: 1.04, 95% CI: 0.93–1.17, *P* = 0.49; *I*^2^ = 86%) or PACG (RR: 0.76, 95% CI: 0.47–1.23, *P* = 0.26; *I*^2^ = 48%; *P* for subgroup difference = 0.21; Figure 2C).

### Association Between Glaucoma and All-Cause Dementia

**Figure 3:**
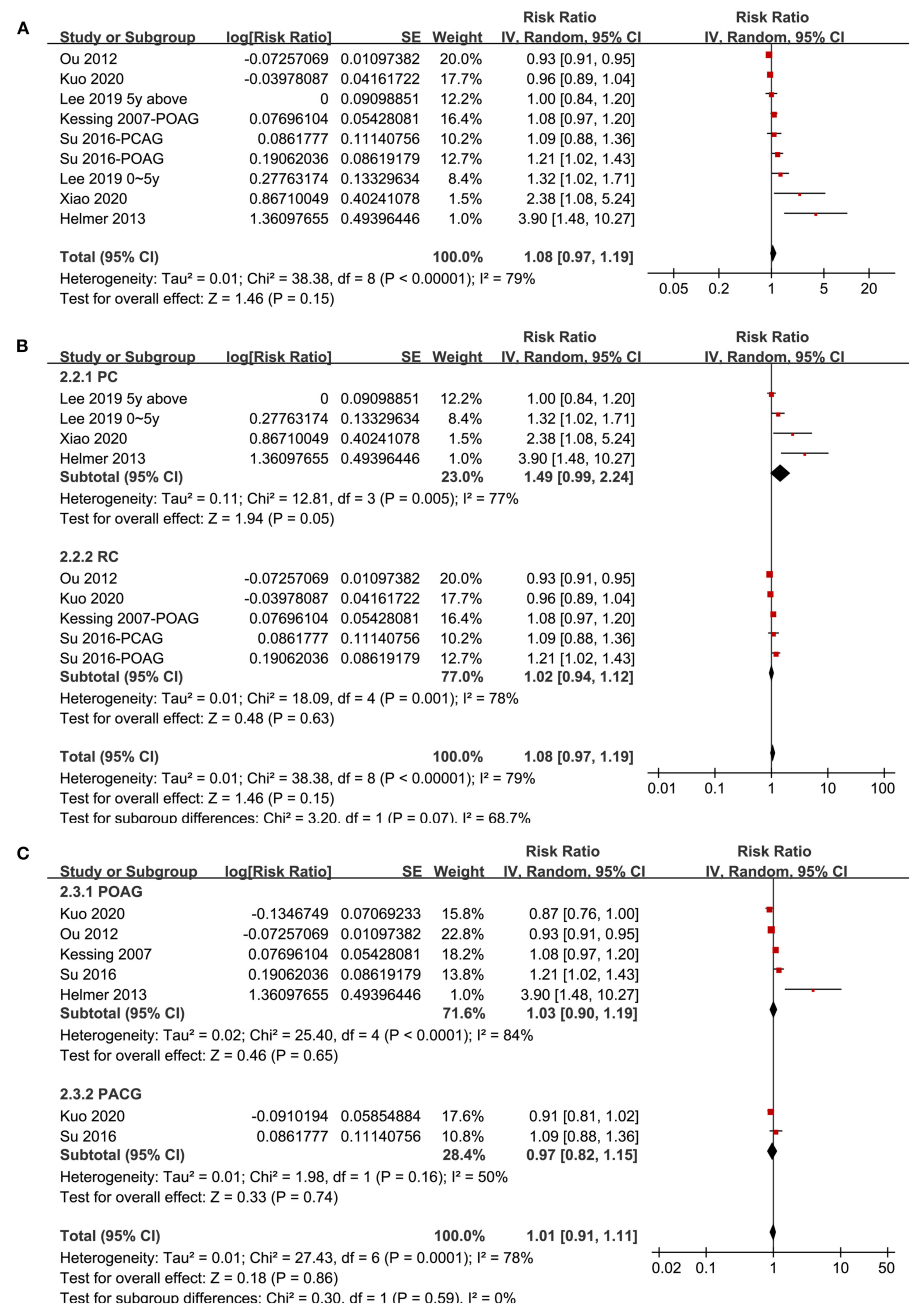
Forest plots for the meta-analysis of the association between glaucoma and the incidence of all-cause dementia in adult population; **(A)** overall meta-analysis; **(B)** subgroup analysis according to the study design; and **(C)** subgroup analysis according to the type of glaucoma.

Seven studies (20–22, 29–31, 34) reported the association between glaucoma and all-cause dementia. One study reported the associations of POAG and PACG separately (34), and the other study reported the associations of recent (diagnosed within 5 years) and established (diagnosed > 5 years) glaucoma (20), and these datasets were included independently. Overall, meta-analysis of nine datasets from seven cohort studies showed that glaucoma was not independently associated with an increased incidence of all-cause dementia (adjusted RR: 1.08, 95% CI: 0.97–1.19, *P* = 0.15; Figure 3A) with significant heterogeneity (*P* for Cochrane's *Q*-test < 0.001, *I*^2^ = 79%). Sensitivity analyses by excluding one study at a time showed consistent results (*P* all > 0.05). Subgroup analyses showed that glaucoma was not independently associated with an increased incidence of AD in prospective (RR: 1.49, 95% CI: 0.99–2.24, *P* = 0.05) or retrospective studies (RR: 1.02, 95% CI: 0.94–1.12, *P* = 0.63; *P* for subgroup difference = 0.07; Figure 3B), or in studies with patients with POAG (RR: 1.03, 95% CI: 0.90–1.19, *P* = 0.65) or PACG (RR: 0.97, 95% CI: 0.82–1.15, *P* = 0.74; *P* for subgroup difference = 0.86; Figure 3C).

### Association Between Glaucoma and Non-Ad Dementia

**Figure 4:**
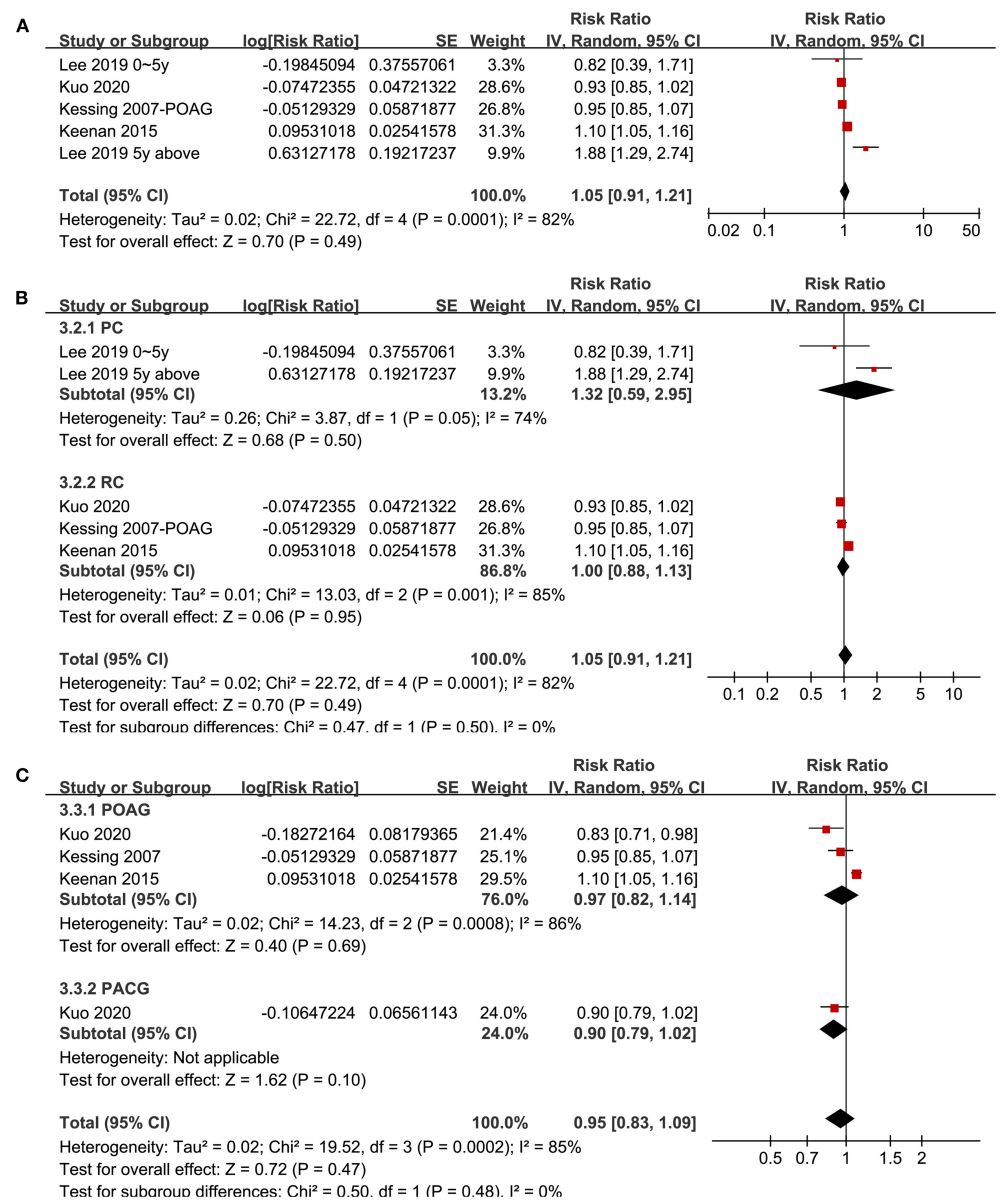
Forest plots for the meta-analysis of the association between glaucoma and the incidence of non-AD dementia in adult population; **(A)** overall meta-analysis; **(B)** subgroup analysis according to the study design; and **(C)** subgroup analysis according to the type of glaucoma.

Four studies (20, 21, 29, 33) with five datasets reported the association between glaucoma and non-AD dementia. Pooled results showed that glaucoma was not independently associated with an increased incidence of all-cause dementia (adjusted RR: 1.05, 95% CI: 0.91–1.21, *P* = 0.49; *I*^2^ = 82%; Figure 4A). Sensitivity analyses by excluding one study at a time showed consistent results (*P* all > 0.05). Subgroup analyses according to the study design and type of glaucoma showed consistent results (*P* for subgroup difference both > 0.05; Figures 4B,C).

### Publication Bias

**Figure 5:**
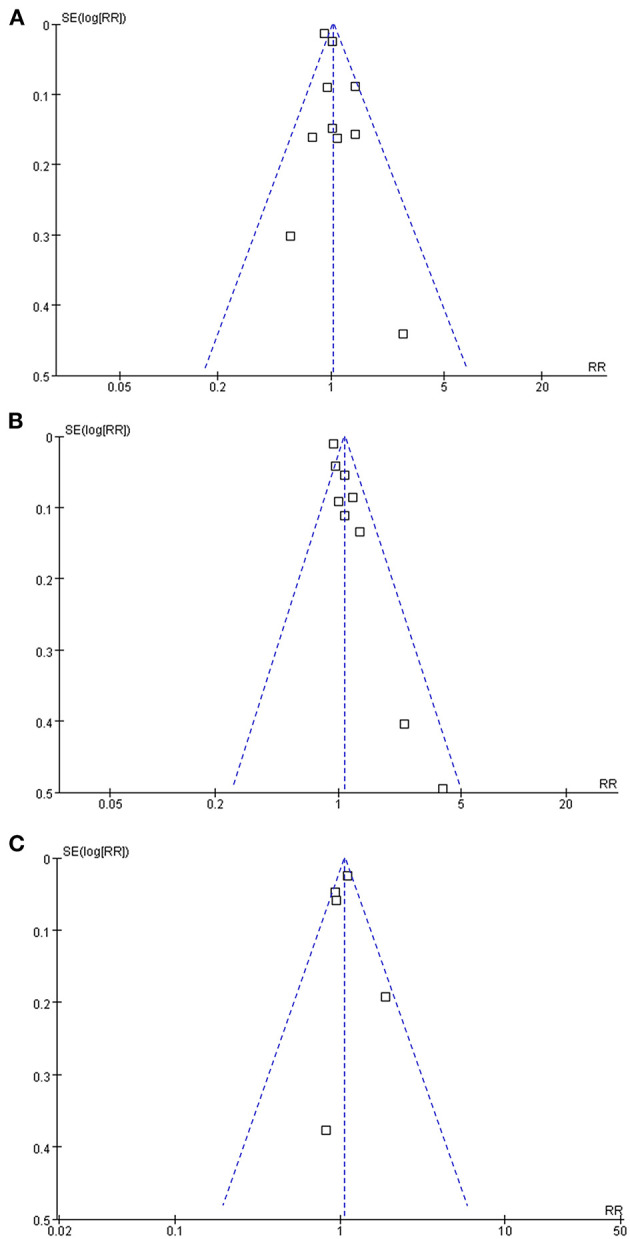
Funnel plots for the publication bias underlying the meta-analyses; **(A)** funnel plots for the meta-analysis of the association between glaucoma and the incidence of AD; **(B)** funnel plots for the meta-analysis of the association between glaucoma and the incidence of all-cause dementia; and **(C)** funnel plots for the meta-analysis of the association between glaucoma and the incidence of non-AD dementia.

The funnel plots regarding the associations between glaucoma with AD, all-cause dementia, and non-AD dementia were shown in Figures 5A–C. The funnel plots were symmetry on visual inspection, suggesting low risk of publication bias. Egger's regression tests also showed no significant publication biases for the meta-analyses of the associations between glaucoma with AD and all-cause dementia (*P* = 0.52 and 0.19, respectively). For the meta-analysis of the association between glaucoma and non-AD dementia, Egger's regression test was unable to be performed since only five datasets were included.

## Discussion

In this study, we pooled the results of up-to-date cohort studies and showed that compared to populations without glaucoma, patients with glaucoma were not associated with higher incidences of AD, all-cause dementia, or non-AD dementia. Subsequent sensitivity analyses and subgroup analyses showed that the results of the meta-analyses were not primarily affected by either of the included study, and the results were not affected by study characteristics such as study design and subtype of glaucoma. Taken together, these findings suggested that although glaucoma and AD shared some common pathophysiological features, current evidence from cohort studies did not support that glaucoma is an independent risk factor for AD, all-cause dementia, or non-AD dementia.

Compared to previous meta-analysis of the similar topic, our study has multiple strengths. Only cohort studies were included in our study since including cross-sectional studies, such as case-control studies may expose the findings to higher risk of patient selection bias and recall bias. Besides, only studies with multivariate adjusted analyses were included in this meta-analysis, which therefore substantially reduced the potential influence of confounding factors on the outcome. Accordingly, our meta-analysis was expected to retrieve a potential independent association between glaucoma and AD and other dementia outcomes. However, by using these strict inclusion criteria, our study failed to indicate a significant association between glaucoma and the incidence of AD, all-cause dementia, and non-AD dementia. In addition, results of sensitivity analyses and subgroup analyses according to study design and subtype of glaucoma showed consistent results, which further confirmed the robustness of the findings. Interestingly, results of our finding are inconsistent with previous cross-sectional studies, which generally showed that glaucoma is associated with higher prevalence of AD, and dementia attributed to other causes, such as vascular dementia (17–19, 37, 38). These divergences may be explained by the differences in methodology between cross-sectional and cohort studies. The appeared connection between glaucoma and AD may be a reflection of the shared demographic, genetic, and pathophysiological features of patients with the two diseases, such as aging, ε4/ε4 genotype of *ApoE*, and deposition of amyloid-β peptides etc. (13, 15). However, as a meta-analysis of cohort studies with multivariate analysis, results of study indicated that for patients with confirmed diagnosis of glaucoma, incidences of subsequent AD, all-cause dementia, and non-AD dementia were not increased as compared to those without the diagnosis of glaucoma, particularly after the adjustment of the potential confounding factors.

Previous studies in transgenic mouse model of AD showed altered processing of amyloid precursor protein and accumulation of β-amyloid peptides in neurons of retinal ganglion cell layer (RGCL) and inner nuclear layer (INL), along with a slightly elevated intraocular pressure (IOP) (39, 40), suggesting the occurrence of optical neurodegeneration during the pathogenesis of AD. It has to be mentioned that although our findings did not support that glaucoma is an independent risk factor for subsequent development of AD, in view of the lack of effective strategies for early screening of AD before the occurrence of memory dysfunction (41), ophthalmologic examinations remain of potential as early screening tools for AD. With the development of novel techniques such as retinal optical coherence tomography (42, 43) and nanomaterial biosensors (44), it could be expected that eye clinics and ophthalmology facilities may play important roles in the early diagnosis of AD and subsequent reducing the burdens arising from severe dementia (45).

Our study has some limitations, too. Firstly, the number of eligible studies in this meta-analysis is small, which prevented the further analyses on the influences of patient characteristics on the outcome, such as age, sex, and comorbidities. A meta-analysis based on individual-patient data, rather than data from study-level, is needed for subsequent investigation. In addition, although studies showed consistent results in patients with POAG and PACG, we were unable to determine whether the results were consistent for patients with different severity of glaucoma (such as those with different levels of IOP). Severe glaucoma which causes visual impairment may lead to dementia via sensory deprivation. Moreover, the mean ages of the participants of the included studies varied from 59 to 80 years. Therefore, the results may mainly reflect the association between glaucoma and risk of dementia in the elderly. Finally, influences of therapies targeting glaucoma on the subsequent risks of AD, all-cause dementia, and non-AD dementia remain unknown. Future studies may be considered for further evaluation.

In conclusion, despite of the overlapped demographic, genetic, and pathophysiological features between glaucoma and AD, results of this meta-analysis did not support that glaucoma is an independent risk factor for AD, all-cause dementia, or non-AD dementia. However, in view of the possible occurrence of optical neurodegeneration during the pathogenesis of AD, ophthalmologic examinations remain of potential as early screening tools for AD.

## Data Availability Statement

The original contributions presented in the study are included in the article/supplementary material, further inquiries can be directed to the corresponding author/s.

## Author Contributions

WZ and JT designed the study and wrote the manuscript. WZ and XL performed literature search, data extraction, and quality evaluation. All authors performed statistical analyses and interpreted the results, reviewed and revised the manuscript, and approved the manuscript for submission.

## Conflict of Interest

The authors declare that the research was conducted in the absence of any commercial or financial relationships that could be construed as a potential conflict of interest.

## Publisher's Note

All claims expressed in this article are solely those of the authors and do not necessarily represent those of their affiliated organizations, or those of the publisher, the editors and the reviewers. Any product that may be evaluated in this article, or claim that may be made by its manufacturer, is not guaranteed or endorsed by the publisher.
